# An Efficient Ambulance Service for London

**Published:** 1913-02-08

**Authors:** 


### AN EFFICIENT AMBULANCE SERVICE FOR LONDON.

### The County Council Blocks the Way.

### UNFULFILLED PROMISES AND INDIFFERENT CANDIDATES.

With a view to secure the prompt enforcement of the Metropolitan Ambulances Act, a letter was addressed before the last County Council election in 1910 to every candidate by the founder of the Street Ambulance Service, countersigned by the Honorary Secretary to the Bischoffsheim Ambulances, pointing out that it was not a question of party but of humanity to support the immediate establishment of an efficient ambulance service for London without delay. The candidates whose addresses could be traced consisted of 114 Municipal Reformers and 111 Progressives, or together 225. Of these 71 Municipal Reformers and 71 Progressives, or together 142 candidates, replied, but of these 142 only 63 were elected members. Of these 63 elected members, 25 Municipal Reformers and 28 Progressives declared themselves in favour of the County Council establishing an efficient ambulance service in London, similar to that working in the City under the powers conferred by the Metropolitan Ambulances Act, 1909. The remaining 10 members gave the qualified approval appended to their names on p. 506.

### What will Londoners Do?

Three years have elapsed since the above recorded poll was taken; Greater London is still without an efficient ambulance service, and nothing practical has been done by the London County Council to put in force the Metropolitan Ambulances Act of 1909. We are informed that some Councillors hold that it is their duty not to increase the rates beyond the amount at which they at present stand. This is a view that the ratepayers might be more grateful for, if they did not realise that the cost of an efficient ambulance service is estimated at £15,000 a year, which would mean for the ratepayers something like one-third of a farthing in the £. A penny rate now yields in London, we understand, in round figures £180,000 a year.

The time has certainly come when this question of an efficient ambulance service for London must be taken in hand and dealt with effectually without a moment's avoidable delay. Some Councillors, it will be seen, suggested the Metropolitan Asylums Board might set up a general ambulance service, but in our view it is the distinct duty of the London County Council to undertake this pressing work on behalf of the inhabitants of London, and we are glad to know that the Parliamentary Committee confirmed this view by an overwhelming majority. It has been the fashion for some London County Councillors to belittle the work and efficiency of the Corporation of the City of London. Yet the Corporation of the City of London has set up an ambulance service and has worked it for over three years with perfect success, to the diminution of human suffering and to the saving of human life. It passes our belief that any candidate at the forthcoming election to the London County Council will stand up at the meetings about to be held, and like Pilate, calmly invite the ratepayers' support for himself, when he has used all his influence to injure the reputation of the London County Council by countenancing the neglect of the present Council to settle this ambulance question satisfactorily. We hope that some independent speakers and independent ratepayers, quite apart from party, will make it their business to attend every public meeting during the coming County Council election, to heckle every candidate and compel him to declare whether he will pledge himself to work unceasingly for an efficient ambulance service for London, or not.

### The Past Neglect and the Electors' Opportunity.

We publish below a list showing the attitude taken up on this question by the members of the present Council. We hope that in every division adequate steps will be taken to ' rub in ' the inaction of the councillors, the broken promises and the supineness of those who have not even taken the trouble to express any view on this important question. We make bold to say that no man is fit to serve upon the London County Council who does not realise that it is essential that Greater London shall no longer be left without an adequate, up-to-date, and efficient ambulance service. Further, that the duty of establishing and working this service rests with the London County Council, and with no one else. Bearing in mind the ever-increasing number of serious accidents in the streets of London, this question of adequate ambulance provision is one which every prudent ratepayer should make it his business to press with energy at the present time. **Let no single vote be given at the ensuing election for any candidate of either party who is not pledged if elected to protect the citizens without a moment's further delay from the present dangers and miseries to which every one of them is daily subjected through the past neglect of this question by the retiring members of the present London County Council.**

### County Councillors who Promised Sympathy.

The following members of the present L.C.C. signed a postcard in March 1910 as follows:—" I am in favour of the County Council establishing an efficient Ambulance Service in London, similar to that now working in the City under the powers conferred by the Metropolitan Ambulances Act, 1909."

(Note.—*The letters in parentheses after the name indicate the party*—i.e., *M.R., Municipal Reform; P., Progressive.*)

Battersea.—Walter Warren (P.).
Brixton.—Ernest Gray (M.R.), W. Haydon (M.R.).
Camberwell (North).—R. A. Bray (P.).
City of London.—J. W. Domoney (M.R).

Clapham.—Lord Dunmore (M.R.), R. M. Sebag-Montefiore (M.R.).

Deptford.—E. Mumford Preston (M.R.), W. Freeman Barrett (M.R.).

Finsbury (Central).—Arthur B. Russell (P.).

Finsbury (East).—H. E. A. Cotton (P.), G. M. Gillett, J.P. (P.).

Hackney (Central).—Miss N. Adler (P.), Alderman A. J. Shepheard (P.).

Hackney (North).—O. Warburg (M.R.), G. W. H. Jones (M.R.).

Hackney (South).—T. Chapman (P.).

Haggerston.—Stephen Gee (P.).

Hampstead.—W. Reynolds (M.R.).

Islington (East).—E. Smallwood (P.).

Islington (North).—J. C. Hill (M.R.) (since resigned).

Islington (South).—George Dew (P.).

Islington (West).—R. C. Lambert, J.P. (P.).

Kennington.—Baron de Forest (P.).

Lambeth (North).—F. Briant (P.), F. S. Smith (P.).

Lewisham.—F. Carter (M.R.), Earl Stanhope (M.R.).

Limehouse.—A. W. Yeo, J.P. (P.).

Marylebone (East).—Col. H. A. Pakenham (M.R.) (since resigned).

Marylebone (West).—E. White (M.R.), Miss Susan Lawrence (M.R.) (since resigned).

Mile End.—James May (P.), C. Stettauer (P.).

Norwood.—F. St. John Morrow (M.R.), C. U. Fisher (M.R.).

Paddington (North).—C. E. Goff (M.R.).

Paddington (South).—Major Lewis-Barnes (M.R.).

Peckham.—T. Gautrey (P.), Lord Haddo (P.).

Rotherhithe.—Rev. Dr. J. Scott Lidgett (P.).

St. George's-in-the-East.—Harry Gosling (P.), C. J. Matthew (P.).

St. Pancras (East).—A. W. Claremont (P.), H. C. Lea (P.).

St. Pancras (South).—Sir G. Alexander (M.R.), J. C. Denison Pender (M.R.).

St. Pancras (West).—W. Lloyd Taylor, J.P. (P.).

Walworth.—J. A. Dawes, M.P. (P.), C. Jesson (P.).

Wandsworth.—W. Hunt (M.R.).

Westminster.—R. W. Granville-Smith (M.R.), C. Y. Sturge (M.R.) (since deceased).

### Qualified Approval.

The following County Councillors signed the postcard (or wrote a letter), but with a qualification as indicated:—

Chelsea.—E. L. Meinertzhagen (M.R.). In favour of M.A.B. Ambulance, not L.C.C. Fulham.—Cyril Cobb (M.R.). "As soon as the exigencies of the financial position allow and being assured that no increase in the rates would be entailed." E. G. Easton (M.R.). ?The L.C.C. or Police, Fire Brigade, or M.A.B. Greenwich.—G. H. Hume (M.R.). In favour "in the abstract, but cannot pledge myself before studying the subject more closely." Islington (North).—F. L. Dove (M.R.). "Could not give unconditional support without further detailed inquiries." Islington (West).—Alderman H. Jephson (P.). "Cannot pledge myself as to it being exactly similar to that working in the City." Kensington (North).—Major Skinner (M.R.) (resigned), David Davis (M.R.). Struck out words "similar to that now working in the City." Kensington (South).—Col. Cavaye, J.P. (M.R.). "Will give the subject my most favourable consideration." St. Pancras (North).—T. F. Hobson (P.). "Object excellent, but project new, and shall have to consider it."

### County Councillors Unpledged.

Members of the foregoing divisions who have not expressed an opinion on the Ambulance question:—

A. A. Allen, M.P. (P.).—Haggerston.
Sir J. Benn, J.P. (P.).—Kennington.
W. A. Casson (P.).—South Hackney.
W. Davies, J.P. (P.).—Battersea.
J. H. Hunter (M.R.).—North Paddington.
C. Jackson (M.R.).—Limehouse.
J. B. Karslake (M.R.).—South Paddington.
A. L. Leon, J.P. (P.).—North St. Pancras.
S. Lithgow (P.).—West St. Pancras.
J. W. Lorden (M.R.).—Wandsworth.
R. C. Norman (M.R.).—Chelsea.
W. H. Pannell, J.P. (M.R.).—City of London.
L. W. Rostron (M.R.).—Central Finsbury.
Hon. Chas. Russell (P.).—Bermondsey.
Stuart Sankey (M.R.).—City of London.
R. L. Stuart, J.P. (P.).—Rotherhithe.
A. T. Taylor (M.R.).—Hampstead.
H. R. Taylor, J.P. (P.).—North Camberwell.
A. A. Thomas (P.).—East Islington.
W. W. Thompson (M.R.).—South Kensington.
Lord A. Thynne, M.P. (M.R.).—East Marylebone.
H. J. Williams, J.P. (P.).—South Islington.

### Councillors or Electors for whom this Question has no Interest.

In the following divisions it would appear that neither the members nor the electors had at the last election the slightest apprehension of the importance of an adequate ambulance provision for the whole Metropolis. At any rate not one of the undermentioned members returned the postcards or expressed an opinion on the question. The same remarks apply to those members in other divisions whose names do not appear in the above list.

List of divisions where none of the present members have expressed an opinion on the ambulance question, with their names and parties:—

Bermondsey.—Hon. C. Russell (P.), W. H. Ecroyd (P.).

Bethnal Green (N.E.).—Garnham Edmonds (P.), E. Smith (P.).

Bethnal Green (S.W.).—Rev. S. D. Headlam (P.), P. A. Harris (P.).

Bow and Bromley.—G. Lansbury (Lab.), G. L. Bruce (P.).

Dulwich.—F. Hall (M.R.), Sir A. Griffith-Boscawen (M.R.).

Hammersmith.—J. Brandon (M.R.), I. Salmon (M.R.).

Holborn.—Hon. H. Lygon (M.R.), R. I. Tasker (M.R.).

Hoxton.—B. B. Evans (P.), J. Stanley Holmes (P.).

Newington (West).—James D. Gilbert (P.), Evan Spicer (P.).

Poplar.—Sir John McDougall (P.), R. C. K. Ensor (Lab).

St. George's, Hanover Square.—Lord Cheylesmore (M.R.), H. J. Greenwood (M.R.).

Southwark (West).—Albert Wilson (P.), Thomas Hunter (P.).

Stepney.—A. O. Goodrich (M.R.), J. Sankey, K.C. (M.R.).

Strand.—Lieut-Col. Probyn (M.R.), P. E. Pilditch (M.R.).

Whitechapel.—William C. Johnson (P.), H. H. Gordon (P.).

Woolwich.—W. J. Squires (M.R.).

